# Supplementary material for: Epidemiology of Cognitive Impairments: Demographic and Clinical Predictors of Memory and Attention Challenges—Findings from Twelve National Disability Indicators
Source: J Clin Med. 2025 Sep 10;14(18):6390. doi: 10.3390/jcm14186390 (PMC12470920; doi:10.3390/jcm14186390)
Supplement: Supplementary file 1 [file jcm-14-06390-s001.zip › supplementary file S2.pdf]

Supplementary file S2

Saudi Citizens Reporting Any Functional Difficulty, by Age Group and Sex (2017)

Age group (years)	Total with difficulty n (%)†	Females n (%)‡	Males n (%)‡
0 – 4	26,520 (1.2)	13,387 (1.3)	13,133 (1.2)
5 – 9	47,087 (2.2)	19,157 (1.8)	27,930 (2.6)
10 – 14	59,855 (3.2)	26,123 (2.8)	33,732 (3.5)
15 – 19	54,430 (3.0)	26,568 (3.0)	27,862 (3.1)
20 – 24	61,390 (3.0)	25,844 (2.7)	35,546 (3.4)
25 – 29	61,565 (3.2)	21,881 (2.3)	39,684 (4.1)
30 – 34	69,534 (4.0)	26,798 (3.1)	42,736 (4.8)
35 – 39	73,422 (4.8)	25,118 (3.3)	48,304 (6.2)
40 – 44	81,264 (6.3)	31,772 (5.0)	49,492 (7.6)
45 – 49	84,270 (7.9)	35,885 (6.9)	48,385 (8.8)
50 – 54	115,606 (13.6)	60,200 (14.5)	55,406 (12.6)
55 – 59	122,823 (18.7)	65,787 (21.0)	57,036 (16.7)
60 – 64	137,728 (28.7)	66,960 (28.9)	70,768 (28.6)
65 – 69	122,508 (39.5)	63,911 (40.0)	58,597 (38.9)
70 – 74	106,912 (48.0)	59,204 (52.5)	47,708 (43.4)
75 – 79	85,990 (59.7)	48,114 (66.0)	37,876 (53.2)
80 +	134,819 (76.2)	73,779 (80.6)	61,040 (71.4)
Total	1,445,723 (7.1)	690,488 (6.9)	755,235 (7.3)

† Percentage of each age-group's total Saudi population that reported **any** functional difficulty (all types combined).

‡ Percentages are within-sex proportions for the same age group.

Data source: 2017 Saudi National Disability Survey (public release tables).
