# Supplementary material for: Epidemiology of Cognitive Impairments: Demographic and Clinical Predictors of Memory and Attention Challenges—Findings from Twelve National Disability Indicators
Source: J Clin Med. 2025 Sep 10;14(18):6390. doi: 10.3390/jcm14186390 (PMC12470920; doi:10.3390/jcm14186390)
Supplement: Supplementary file 1 [file jcm-14-06390-s001.zip › supplementary file S3.pdf]

Supplementary file S3

Saudi citizens reporting multiple difficulties by age group and sex, 2017

Age group (y)	Total n (%)	Females n (%)	Males n (%)
0 – 4	11 390 (0.5)	6 428 (0.6)	4 962 (0.5)
5 – 9	18 739 (0.9)	7 850 (0.8)	10 889 (1.0)
10 – 14	16 561 (0.9)	7 429 (0.8)	9 132 (1.0)
15 – 19	17 068 (1.0)	8 032 (0.9)	9 036 (1.0)
20 – 24	20 994 (1.0)	7 308 (0.8)	13 686 (1.3)
25 – 29	19 724 (1.0)	5 366 (0.6)	14 358 (1.5)
30 – 34	15 165 (0.9)	7 832 (0.9)	7 333 (0.9)
35 – 39	19 376 (1.3)	5 955 (0.8)	13 421 (1.9)
40 – 44	22 648 (1.8)	8 679 (1.5)	13 969 (2.3)
45 – 49	22 534 (2.1)	9 489 (2.0)	13 045 (2.6)
50 – 54	39 618 (4.6)	22 008 (6.2)	17 610 (4.6)
55 – 59	48 458 (7.4)	29 249 (11.8)	19 209 (6.7)
60 – 64	57 427 (12.0)	26 070 (15.8)	31 357 (17.7)
65 – 69	70 535 (22.7)	36 329 (38.0)	34 206 (37.1)
70 – 74	65 743 (29.5)	36 888 (68.8)	28 855 (26.2)
75 – 79	59 929 (41.6)	32 633 (44.7)	27 296 (38.4)
80 +	106 169 (60.0)	60 186 (65.7)	45 983 (53.8)
Total	632 078	317 731	314 347

(percentages are the share of each age-group's population who reported ≥2 functional difficulties; row percentages are shown in parentheses)
